# Association between periodontal disease and plasma levels of cholesterol and triglycerides

**Published:** 2013-06-30

**Authors:** Adriana Jaramillo, Gloria Inés Lafaurie, Lina Viviana Millán, Carlos Martin Ardila, Andrés Duque, Camilo Novoa, Diego López, Adolfo Contreras

**Affiliations:** a Periodontal Medicine Group, School of Dentistry, Universidad del Valle. E-mail: adolfoco@yahoo.com; b Instituto UIBO, Faculty of Dentistry, Universidad El Bosque. E-mail: institutouibo@gmail.com; c Universidad de Antioquia, Medellin Colombia. E-mail:martinardila@gmail.com; d Faculty of Dentistry, Universidad CES. E-mail: aduqued@ces.edu.co; e Faculty of Dentistry, Pontificia Universidad Javeriana. E-mail: novoacamilo@hotmail.com; f School of Dentistry, Universidad del Valle. E-mail: dilote@hotmail.com

**Keywords:** Periodontal disease, dyslipidemia, HDL, LDL, triglycerides

## Abstract

**Objective:**

untreated periodontal disease seems to cause low grade systemic inflammation and blood lipid alteration leading to increased cardiovascular disease risk. To start testing this hypothesis in colombian patients, a multicentre study was conducted including the three main state capitals: bogota, medellin and cali.

**Methods:**

in this study 192 (28.4%) advanced and 256 (37.8%) moderate periodontitis patients were investigated for socio-demographic variables, city of precedence, periodontal parameters, smoking, red complex periodontopathic bacteria, serum antibodies against *porphyromonas gingivalis* and *aggregatibacter actinomycetemcomitans* and blood lipids including total cholesterol, hdl, ldl and triglycerides (tg). Those parameters were compared to 229 (33.8%) controls having periodontal health or gingivitis.

**Results:**

advanced periodontitis had worst periodontal indexes, than moderate periodontitis and controls. Interestingly, higher hdl and tg levels were present in periodontitis. Bmi <30 and smoking were associated with increased hdl, hdl-35, ldl and tg, while glycemia >100 mg/dl associated with hdl, hdl-35 and tg. *Tannerella forsythia* showed a significant association with hdl-35 in bivariate analysis and serum igg1 against *p. Gingivalis* associated with hdl-35 and serum igg1 against *t. Forsythia* associated with tg and serum igg2 against *a. Actinomycetemcomitans* correlated with levels of hdl y hdl-35. In logistic regression the periodontitis patients from cali presented reduced hdl levels as compared to bogota and medellin patients. Presence of igg1 antibodies against *p. Gingivalis* and *a. Actinomycetemcomitans* correlated with reduced hdl levels.

**Conclusion:**

this study confirmed that untreated periodontitis generates alteration in serum lipid levels and systemic bacterial exposure against important periodontopathic bacteria could be the biological link.

## Abstract (es)

**Objetivo:**

La periodontitis no tratada parece causar inflamación sistémica, así como alteración de los niveles sanguíneos de lípidos, lo que conduce a un mayor riesgo de enfermedades cardiovasculares. Para empezar a probar esta hipótesis en pacientes Colombianos, se realizó un estudio multicéntrico que incluyó las tres ciudades principales: Bogotá, Medellín y Cali.

**Métodos:**

Se estudiaron 192 pacientes con periodontitis avanzada (2.,4%) y 256 (37.8%) con periodontitis moderada, para medir variables sociodemográficas, ciudad de procedencia, parámetros periodontales, fumar, presencia de bacterias periodontopáticas, anticuerpos séricos contra *Porphyromonas gingivalis* y *Aggregatibacter actinomycetemcomitans*, así como niveles de lípidos en sangre incluyendo colesterol total, HDL, LDL y triglicéridos (TG). Estos parámetros se compararon con 229 (33.8%) pacientes controles sanos/gingivitis.

**Resultados:**

Los pacientes con periodontitis avanzada tuvieron peores índices periodontales que los de periodontitis moderada y los controles. Mayores niveles de HDL y TG estuvieron presentes en pacientes con periodontitis. El índice de masa corporal >30 y el hábito de fumar se asociaron con aumento de HDL, HDL-35, LDL y TG, mientras la glicemia >100 mg/dL se asoció con HDL, HDL-35 y TG. En el análisis bivariado *Tannerella forsythia* mostró asociación significativa con HDL-35 e IgG1 sérica contra *P. gingivalis* estuvo asociada a HDL-35 así como IgG1 contra *T. forsythia* con TG y la IgG2 contra *A. actinomycetemcomitans* se correlacionó con los niveles de HDL y HDL-35. En la regresión logística se observó que la región de Cali tuvo niveles menores de HDL en comparación con los pacientes de Bogotá y Medellín. La presencia de anticuerpos IgG1 contra *P. gingivalis* y *A. actinomycetemcomitans* se asoció con niveles reducidos de HDL.

**Conclusión:**

Este estudio confirmó que la periodontitis no tratada genera alteración en los niveles de lípidos séricos y la exposición bacteriana sistémica a las bacterias periodontopáticas podría ser el vínculo biológico.

## Introduction

Periodontitis is a chronic infection characterized by an exaggerated gingival inflammatory response to pathogen microbiota, which results in the loss of dental support tissue and, eventually, in the loss of teeth^1^; it is associated to other systemic chronic conditions like atherosclerotic cardiovascular disease through common pathophysiological pathways, hence, it may be considered that by improving periodontal health local and systemic inflammation is reduced and, thus, cardiovascular risk^2^ may be reduced. In Colombia, according to the results from the most recent National Study on Oral Health in 1999 (ENSAB III), it was found that in the Colombian adult population 50.2% of the people present loss of periodontal insertion and this prevalence increases with age^3^.

Also, cardiovascular diseases are the first causes of death globally, and the WHO estimates that close to 17,3 million people died due to this cause in 2008, which represents 30% of all deaths worldwide^4^.

The foundation of the association between periodontal disease and other systemic inflammatory conditions is chronic inflammation, given that evidence exists that individuals with periodontitis have greater risk of presenting endothelial dysfunction and cardiovascular diseases. The pathogeny of destructive periodontal disease and atherosclerotic disease, hence, can be related through common inflammatory cascades^5^. Among the inflammatory mediators associated to the risk of cardiovascular events we find serum amyloid A, sICAM-1, IL-6, homocysteine, total cholesterol, LDL cholesterol, and C-reactive protein^6^ ^,^ ^7^, which increase in patients with periodontitis inducing pro-coagulation and alterations in lipid metabolism, which can increase risks of cardiovascular events.

The relationship between periodontitis and dyslipidemia seems to a two-way relation, that is, it is not clear if periodontal disease affects lipid metabolism or if the conditions associated to dyslipidemia damage the dental support tissue^8^.

Although it has been suggested that alterations in lipid metabolism and periodontitis may be associated through common physiopathological mechanisms, which explains increased risk of cardiovascular disease in patients with periodontitis^9^ ^,^ ^10^, no published evidence exists on the association between periodontitis and dyslipidemia in Colombia. Due to these reasons, this research sought to determine the association between dyslipidemia and untreated periodontitis from a sample of the Colombian population.

## Materials and Methods

An observational study was carried out with a sample of 677 patients who attended the dental clinics at Universidad El Bosque and Pontificia Universidad Javeriana in Bogota, Universidad CES and Universidad de Antioquia in Medellin, and Universidad del Valle in Cali, during the period comprised between January 2009 and March 2012. The sample size was calculated with a 5% alpha error and 80% beta error to find associations between periodontal risk factors and dyslipidemia with an expected alteration probability ≥15% in lipid profile parameters evaluated in the control population, for an OR ≥2.

### Selection criteria.

 The study included the subjects who accepted their voluntary participation and who attended the dental clinics at the participating universities. Inclusion criteria for patients with periodontitis included having any degree of severity from moderate to severe and at least 14 teeth present in the mouth. The group of control patients (healthy or with gingivitis) was constituted by patients who had maximum four sites with 4 mm pockets in all the sites examined. The study excluded individuals who had received periodontal treatment or systemic antibiotics six months prior to the periodontal exam, and pregnant women or those individuals presenting systemic diseases like HIV or AIDS, cancer, risk of infectious endocarditis and autoimmune diseases.

### Survey to collect information.

Via a structured survey, previously validated through a pilot study, we obtained data corresponding to variables of age, geographic region, gender, socioeconomic level, and smoking habits.

### Periodontal clinical evaluation.

The clinical exam was performed by a periodontist in each of the participating universities, who was trained and calibrated before the start of the study. The exam was conducted of the full mouth, in six sites per tooth, using a periodontal probe UNC 15. The indices obtained were the Loe & Silness gingival index (GI), the plaque index (PI), pocket depth (PB), clinical insertion level (CIL), and bleeding on probing (BOP).

According to the disease severity, patients were distributed into two categories according to the average of the insertion level of the affected sites: slight to moderate chronic periodontitis and severe periodontitis. The control group was made up of patients diagnosed as healthy or with gingivitis.

### Evaluation of periodontal infection indicators.

#### Sample of subgingival plaque.

After the clinical evaluation, the six deepest subgingival sites (pockets>5 mm) were taken in patients with periodontitis and one site per sextant healthy patients; the supragingival plaque was eliminated with a sterile curette and the sample taking site was isolated. Sterile absorbing paper tips were inserted during 20 seconds; these were collected in a sterile Eppendorf tube for their processing in two microbiology laboratories using standardized Polymerase Chain Reaction (PCR) methods.

#### Polymerase Chain Reaction.

Polymerase Chain Reaction was carried out according to the protocol by Ashimoto *et al*., in 1996 and Saiki *et al*., in 1988, with a final reaction volume of 25 µL of which 5 µL corresponded to the sample and 20 µL to the reaction mixture. For *Porphyromonas gingivalis*, *Tannerella forsythia*, and *Treponema denticola*, the raction mix was composed of buffer PCR 1X u 50 mM KCl, 10 mM Tris-HCl (pH 9.0 at 25 °C), 1.5 mM MgCl_2_ and 0.1% Triton (R) X-100, 0.25 U of Taq DNA polymerase, 1.5 mM of MgCl_2_, 0.2 mM from each deoxyribonucleotide and 2 µM from each primer. For *Aggregatibacter actinomycetemcomitans*, the reaction mix was composed of buffer PCR 1X (50 mM KCl, 10 mM Tris-HCl (pH 9.0 at 25 °C), 1.5 mM MgCl_2_ and 0.1% Triton (R) X-100), 0.25 U of Taq DNA polymerase, 2.25 mM of MgCl_2_, 0.2 mM of each deoxyribonucleotide and 2 µM of each primer.

Sample amplification was carried out in a thermocycler (MyCycler Termal Cycler, Bio-Rad). The temperature cycles for *P. gingivalis*, *T. forsythia*, and *T. denticola* included an initial denaturing step at 95 °C for 2 min, followed by 36 denaturing cycles at 95 °C for 30 seconds, alignment at 60 °C for 1 min, extension at 72 °C for 1 min and a final passage at 72 °C for 2 min. The temperature for *A. actinomycetemcomitans* included an initial passage at 95 °C for 2 min, followed by 36 cycles at 94 °C for 30 seconds, 55 °C for 1 min, 72 °C for 2 min, and 72 °C for 10 min.

The PCR products were evaluated via electrophoresis in agarose gel at 1.5% in TAE buffer (Tris-acetate EDTA) stained with 0.5 mg/mL of ethidium bromide and were visualized via trans-illumination with 300 nm UV light. As positive control, we employed DNA from reference strains ATCC 33277 from *P. gingivalis*, ATCC 43037 from *T. forsythia*, ATCC 29522 from *A. actinomycetemcomitans*, and ATCC 35405 from *T. denticola*. As negative control, we employed sterile bi-distilled water. The finding of *P. gingivalis* in the samples was evidenced by the presence of a band corresponding to 404 bp, *T. forsythia* at 641 bp, *T. denticola* at 316 bp, and *A. actinomycetemcomitans* at 557 bp, compared to a marker with 1 Kb molecular weight.

#### Antibody levels against periodontal pathogens

Levels of serum antibodies were conducted at the reference centers, where these methods were previously standardized to assess levels of serum antibodies for the four microorganisms studied. The technique used was indirect ELISA, covering 96-well plates (Immulux Dynex(r)) with *P. gingivalis*, *T. forsythia*, *T. denticola*, and *A. actinomycetemcomitans* homogenized at 2 µg/mL concentration incubated overnight at 4 °C. The specific bonding sites were blocked with 200 °L of albumin at 1% in PBS-Tween (phosphate salt buffer, pH 7.4; Tween 0.05%) at 37 °C for 2 hrs.

Sera from patients were diluted in blocking solution (1:1000) and incubated at 37 °C for 1 hr. Thereafter, the plates were incubated with diluted biotinylated 1:1000 antihuman IgG1 antibody or antihuman IgG2 (Sigma(r)) at 37 °C for 1 hr. Diluted 1:1000 streptavidin-peroxidase conjugate (Vector(r)) was added and incubated at room temperature for 1 hr.

The plates were developed with OPD substrate (O-Phenylenediamine) for 10 min and the reaction was halted with H_2_SO_4_ 2.5 M, to proceed to its reading at 490 nm in a Stat Fax 2100 ELISA reader. To determine IgG1 and IgG2 levels in the samples, specific for each of the microorganisms, standard reference sera were used presenting high levels of IgG1 or IgG2, at different dilutions whose optical density values were plotted on a linear regression curve.

Levels of IgG subtypes in the samples were calculated from the formula Y= M * X + C, obtained with two standard reference sera, and reported as concentration in ELISA Units (EU)/mL. The specific bonds of the assay were determined by conducting ELISA without serum sample and replacing it with water, to subtract this value for each of the serum samples analyzed, and each sample was analyzed in triplicate.

#### Evaluation of lipid profile

Blood samples were taken from each patient via venipuncture, and serum lipid profile was analyzed including total cholesterol, LDL, HDL, and triglycerides (TG). Tests were conducted in the same national level reference laboratory.

### Statistical Analysis

A database was created on ACCESS(r) 2007, which was evaluated periodically using the SAS(r) statistical package. Stata(r) version 9.1. Then, the database was imported to the Stata program version 11.2, to perform the descriptive, bivariate, and multivariate analysis.

Logistic regression models were developed to establish the association of the independent variables with the presence and severity of periodontitis (severity based on insertion level), as well as with the level of inflammatory activity in relationship with pocket depth, level of bleeding, and periodontal infection markers adjusted for age, gender, income level, and geographic region.

Serum was evaluated for levels of total cholesterol, TG, HDL, and LDL among the different groups of healthy/gingivitis patients, with moderate periodontitis and with severe periodontitis, adjusting for confounding factors. The cutoff points to consider lipid levels as risk factor were for HDL cholesterol for cardiovascular risk >40 mg/dL in women and >50 mg/dL in men and for high cardiovascular risk HDL-35 >35 mg/dL. For triglycerides, the cutoff point was 150 mg/dL, for LDL cholesterol <130 mg/dL, and for total cholesterol <200 mg/dL.

## Results

A total of 448 patients with periodontitis were detected in the study of which 192 (28.4%) had advanced periodontitis and 256 (37.8%) moderate periodontitis. The risk factors mentioned were measured in them like socio-demographics, periodontal parameters, smoking, presence of periodontopathic organisms, and serum antibodies against these pathogens, as well as systemic inflammation factors. These groups of patients were compared between periodontitis and against 229 (33.8%) periodontally healthy with gingivitis controls.

Medellin city contributed the highest number of cases of advanced periodontitis with 88 cases (45.8%), followed by Bogotá with 73 (38%), while Cali only had 31 cases (16.1%). Patients with advanced and moderate periodontitis were on the average older (48 yrs) than the control patients (45.5 yrs); this difference was significant (Table 1) with females predominating.

**Table 1:**
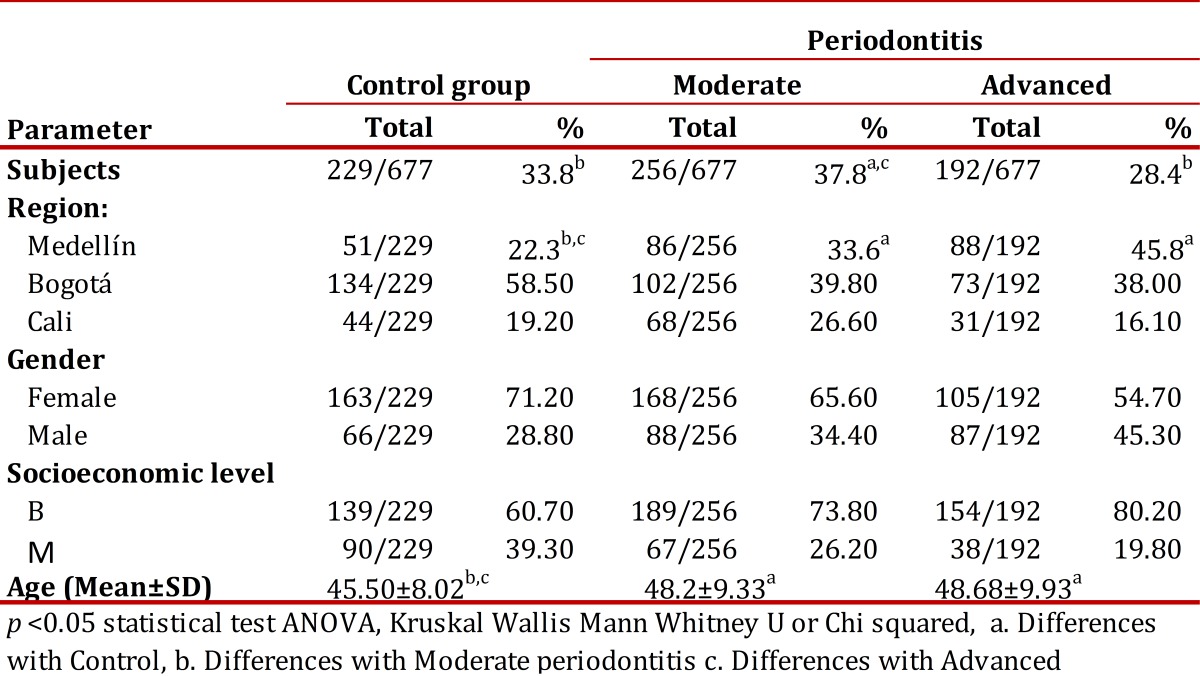
Socio-demographic variables of study subjects

Table 2 evidences that control patients had better periodontal clinical parameters than patients with moderate and advanced periodontitis, given that significant differences were found in average pocket depth, clinical insertion level, bleeding on probing, and in gingival index. Loss of clinical insertion was of 5 ± 1.1 mm in advanced periodontitis, 2.8 ± 0.6 mm in moderate, and 1.3 ± 0.6 mm in the control group.

**Table 2:**
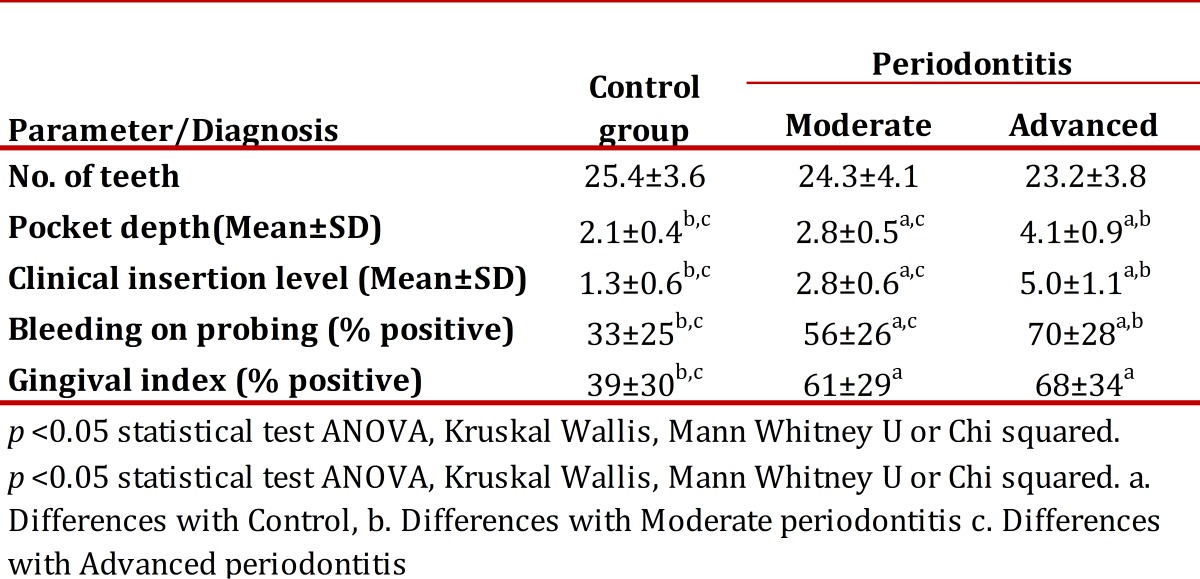
Clinical parameters according to periodontal diagnosis.

Table 3 presents the bivariate analysis correlating socio-demographic variables, periodontal, systemic, environmental and microbiological factors against lipid profiles in these patients. Statistically significant differences were noted among region for total cholesterol, HDL, HDL-35, and TG. Additionally, an association was found among age and total cholesterol and LDL value. The periodontal clinical variables, were associated to cholesterol and LDL levels; however, those differences were not associated to the periodontal clinical state given that controls revealed higher levels of total cholesterol and LDL than patients with periodontitis. The BMI >30 is associated to cholesterol, HDL, HDL-35, LDL, and TG, while glycemia >100 mg/dL is associated to HDL, HDL-35, and TG. Finally, smoking is associated to levels of HDL, HDL-35, and LDL for increased cardiovascular risk.

**Table 3.**
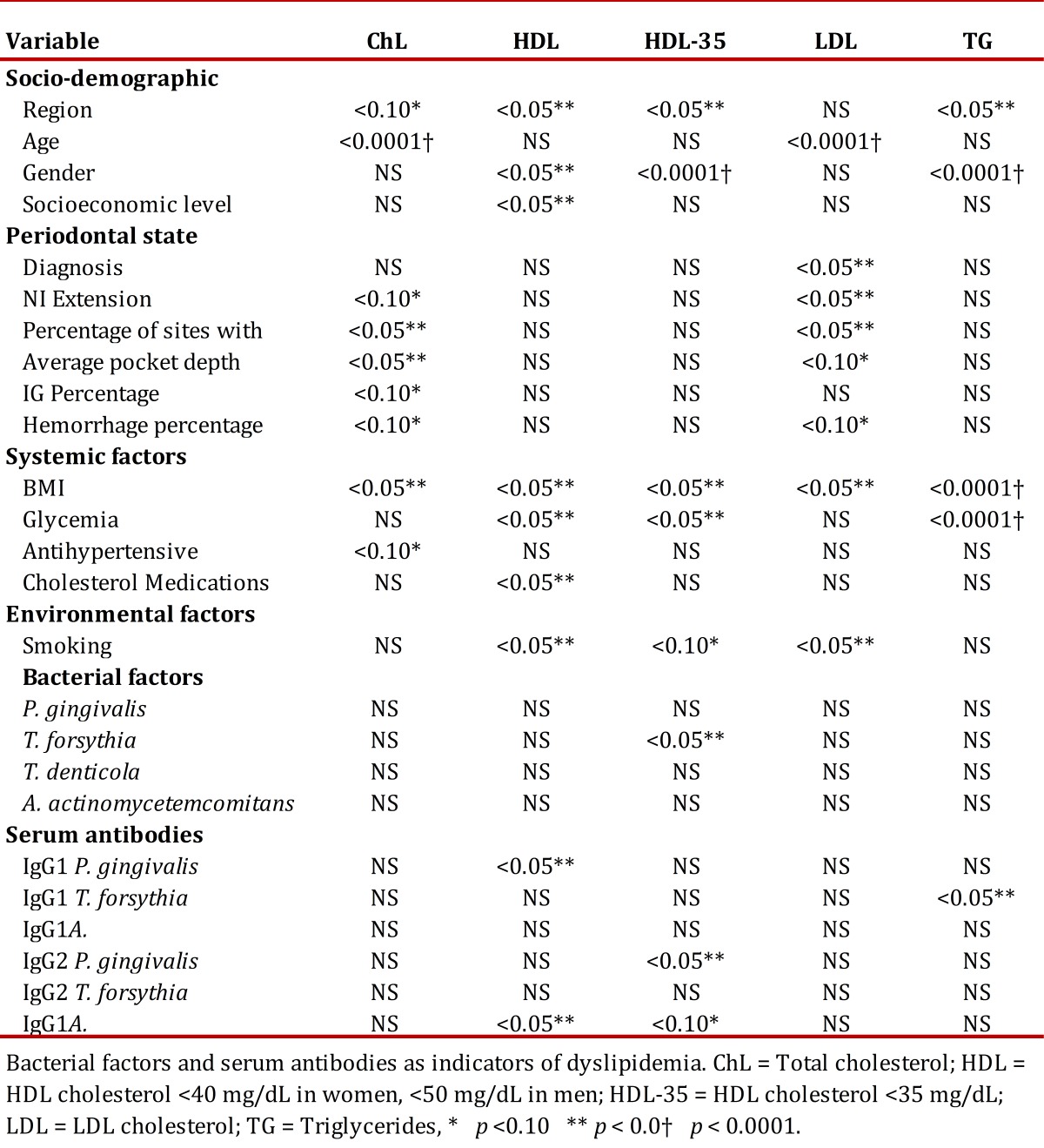
Bivariate analysis of socio-demographic factors, periodontal state, systemic, environmental factors.

Of the microorganisms studied, only *T. forsythia* had a significant association with HDL-35 in the bivariate analysis. With respect to IgG1 serum against *P. gingivalis*, it is associated to HDL-35 and IgG1 serum against *T. forsythia* is associated to TG (Table 3). Additionally, the presence of IgG2 antibodies against *A. actinomycetemcomitans* was correlated to high levels of HDL and HDL-35.

Table 4 presents the risk factors adjusted in the logistic regression. The region of Cali was constituted as a protective factor for HDL levels of medium cardiovascular risk. Having high levels of IgG1 antibodies against *P. gingivalis* and medium and high levels for *A. actinomycetemcomitans* increases the risk of having low HDL levels. Regarding HDL levels that indicate high cardiovascular risk, the associated variables were overweight, glycemia >100 mg/dL, IgG2 for *A. actinomycetemcomitans*, and smoking.

**Table 4:**
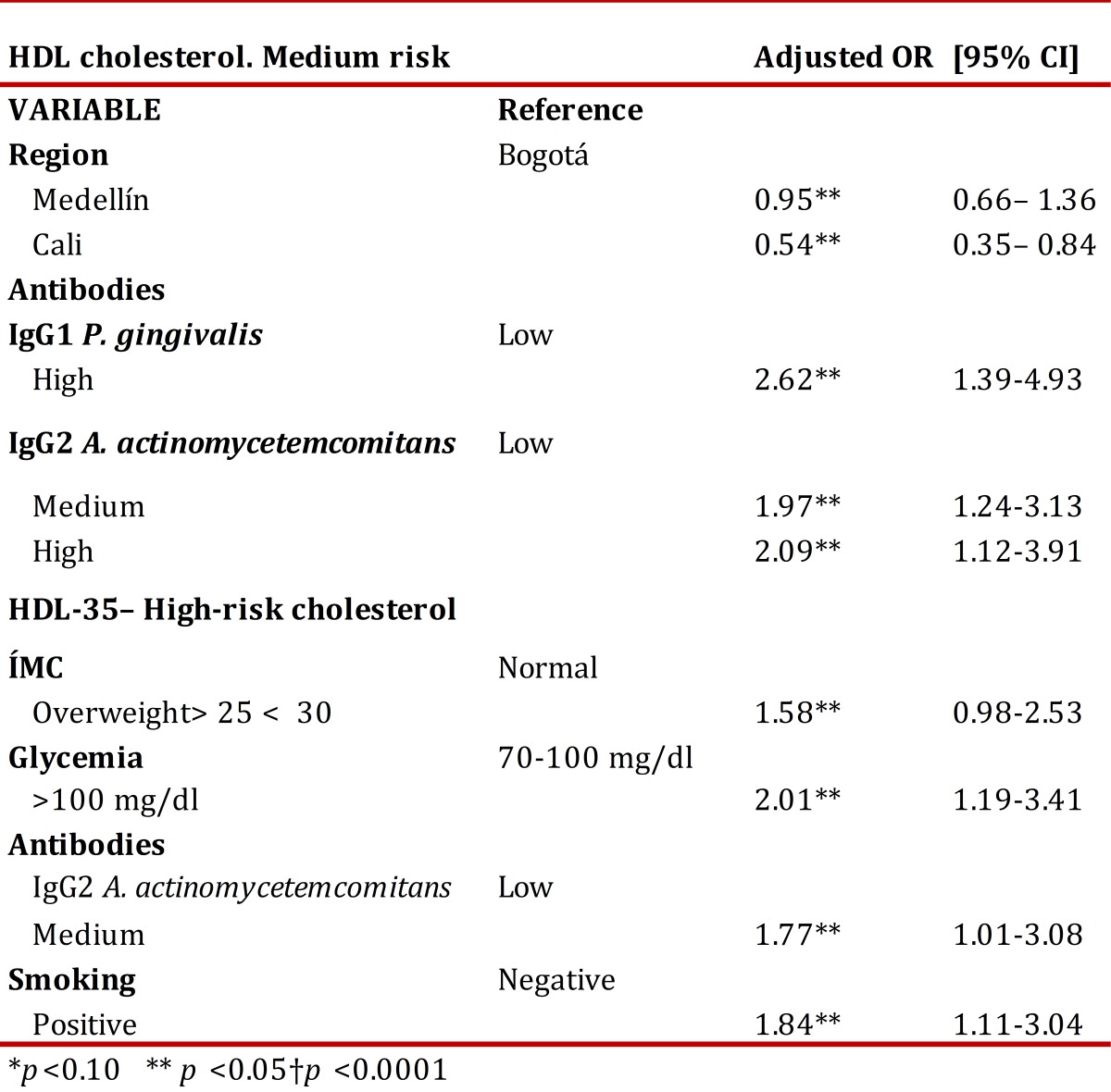
Logistic regression. Risk factors for HDL cholesterol.

## Discussion

Periodontitis has been associated to hypercholesterolemia and hypertriglyceridemia; however, its effects on lipid metabolism are barely known, which is why further laboratory research is required along with intervention clinical studies to treat periodontal diseases to reveal the exact mechanisms that relate periodontitis with dyslipidemia and atherosclerosis. Evidence until now suggests that oral hygiene and periodontal diseases are associated to alteration in lipid metabolism. For example, a study with adult subjects participating on a cohort in Japan found that those self-reporting better tooth-brushing habits had lower levels of triglycerides^11^.

Our observational study revealed that untreated periodontitis is associated to the alteration of important lipid markers related to cardiovascular disease. Patients with periodontitis have lower levels of high-density lipoprotein (HDL) or anti-atherogenic lipoprotein; however, this is not associated to the unexpected increase of low-density lipoprotein (LDL) or pro-atherogenic lipoprotein (Table 3). Other researchers have found association between alterations in lipid metabolism and periodontitis. In a population-based study in Korea, the authors reported adjusted ORs of 1.38 (95%CI: 1.17-1.62) for hypertriglyceridemia and 1.34 (95%CI: 1.14-1.56) for low high-density lipoprotein cholesterol^12^. Also, in the United States, an adjusted OR was found between moderate periodontitis and low levels of HDL at 1.422. In Brazil, in a study of cases and controls no associations were found between the severity of the periodontitis and the lipid serum levels, possibly due to the limited size sample and the procedure to select the subjects^13^.

The HDL lipoprotein is considered anti-atherogenic because it neutralizes LPS in the circulation^14^ and prevents LDL oxidation^15^, as well as antagonizes cholesterol transport^16^, because it accepts cholesterol from the cell membrane during its elimination. This process is facilitated by passive diffusion of cholesterol toward HDL and actively by the interaction of lipoproteins poor in apolipoprotein A1 (ApoA1), preBeta-HDL or ABCA1, which facilitates cholesterol removal^17^. HDL cholesterol is esterified in the blood circulation and it is directly transported to the liver via LDL for excretion. Thus, HDL promotes elimination of cholesterol and a failure in this elimination route may be related to thickening of the vascular wall and to the appearance of early atherosclerosis-type lesions in blood vessels^18^.

During chronic inflammation, as in the case of untreated periodontitis, changes occur in lipoprotein distribution and in the proportions of cholesterol subtypes^19^. Infection increases HDL catabolism and can reduce levels of HDL cholesterol^20^. Thus, the main protein, ApoA1, is displaced by an increase in lipid serum amyloid A (SAA), which is synthesized in big proportion in response to an increase in pro-inflammatory proteins^21^. Triglycerides also increase when there is inflammation and infection, phenomena in which cholesterol- and HDL-rich complexes are formed, and are substrate for hepatic lipase that when activated stimulates the formation of poor lipids that suffer accelerated catabolism through the kidney^22^. Within this scenario, infection and inflammation cause dramatic changes in HDL levels and in its metabolism^23^. 

Infection also induces some other atherogenic changes in lipoprotein profiles and these can be some of the mechanisms that link chronic inflammation to the development of atherosclerosis. Various pathogens are capable of causing alterations in lipid metabolism, among them are *Chlamydia pneumoniae*, *Helicobacter pylori* ^24^, and periodontal pathogens *P. gingivalis*, *T. forshytia*, and *A. actinomycetemcomitans* that have been found associated to diverse lipid metabolites^25^ ^,^ ^26^. With respect to the level of antibodies to the periodontal pathogens, in the present study, *T. forsythia* had a significant association with HDL-35 in the bivariate analysis (Table 3), while Serum IgG1 against *P. gingivalis* was associated to HDL-35 and Serum IgG1 against *T. forsythia* was associated to TG (Table 3). Additionally, the presence of IgG2 antibodies against *A. actinomycetemcomitans* was correlated to high levels of HDL and HDL-35. However, as shown in table 4 the logistic regression analysis.

Given the temporality of the present study, it may not be ignored that alterations in lipid metabolism occur due to a cause different from periodontitis, but after the adjustment, these variables are associated on the regression model.

In conclusion, this study confirmed that untreated periodontitis is associated to possibly alterations of lipid metabolism; additionally, it was shown that systemic exposure exists to periodontopathic microorganisms, as revealed by the levels of IgG antibodies against *P. gingivalis* and *A. actinomycetemcomitans* associated to increased cardiovascular risk in the regression model. More research is required on this suject to confirm the hypothesis that untreated periodontitis alters lipid metabolism via infection and inflammation.
